# Carotid Body Function in Tyrosine Hydroxylase Conditional Olfr78 Knockout Mice

**DOI:** 10.1093/function/zqae010

**Published:** 2024-02-22

**Authors:** Olalla Colinas, Peter Mombaerts, José López-Barneo, Patricia Ortega-Sáenz

**Affiliations:** Instituto de Biomedicina de Sevilla (IBiS), Hospital Universitario Virgen del Rocío/CSIC/Universidad de Sevilla, Sevile 41013, Spain; Departamento de Fisiología Médica y Biofísica, Facultad de Medicina, Universidad de Sevilla, Seville 41009, Spain; Centro de Investigación Biomédica en Red sobre Enfermedades Neurodegenerativas (CIBERNED), Sevile 41013, Spain; Max Planck Research Unit for Neurogenetics, Frankfurt 60438, Germany; Instituto de Biomedicina de Sevilla (IBiS), Hospital Universitario Virgen del Rocío/CSIC/Universidad de Sevilla, Sevile 41013, Spain; Departamento de Fisiología Médica y Biofísica, Facultad de Medicina, Universidad de Sevilla, Seville 41009, Spain; Centro de Investigación Biomédica en Red sobre Enfermedades Neurodegenerativas (CIBERNED), Sevile 41013, Spain; Instituto de Biomedicina de Sevilla (IBiS), Hospital Universitario Virgen del Rocío/CSIC/Universidad de Sevilla, Sevile 41013, Spain; Departamento de Fisiología Médica y Biofísica, Facultad de Medicina, Universidad de Sevilla, Seville 41009, Spain; Centro de Investigación Biomédica en Red sobre Enfermedades Neurodegenerativas (CIBERNED), Sevile 41013, Spain

**Keywords:** carotid body, glomus cells, chemoreception, oxygen sensing, olfactory receptor, Olfr78 knockout, hypoxic ventilatory response, neuroblasts, responsiveness to hypoxia

## Abstract

The *Olfr78* gene encodes a G-protein-coupled olfactory receptor that is expressed in several ectopic sites. *Olfr78* is one of the most abundant mRNA species in carotid body (CB) glomus cells. These cells are the prototypical oxygen (O_2_) sensitive arterial chemoreceptors, which, in response to lowered O_2_ tension (hypoxia), activate the respiratory centers to induce hyperventilation. It has been proposed that Olfr78 is a lactate receptor and that glomus cell activation by the increase in blood lactate mediates the hypoxic ventilatory response (HVR). However, this proposal has been challenged by several groups showing that Olfr78 is not a physiologically relevant lactate receptor and that the O_2_-based regulation of breathing is not affected in constitutive Olfr78 knockout mice. In another study, constitutive Olfr78 knockout mice were reported to have altered systemic and CB responses to mild hypoxia. To further characterize the functional role of Olfr78 in CB glomus cells, we here generated a conditional *Olfr78* knockout mouse strain and then restricted the knockout to glomus cells and other catecholaminergic cells by crossing with a tyrosine hydroxylase-specific Cre driver strain (TH-Olfr78 KO mice). We find that TH-Olfr78 KO mice have a normal HVR. Interestingly, glomus cells of TH-Olfr78 KO mice exhibit molecular and electrophysiological alterations as well as a reduced dopamine content in secretory vesicles and neurosecretory activity. These functional characteristics resemble those of CB neuroblasts in wild-type mice. We suggest that, although Olfr78 is not essential for CB O_2_ sensing, activation of Olfr78-dependent pathways is required for maturation of glomus cells.

## Graphical Abstract

**Graphical Abstract:**
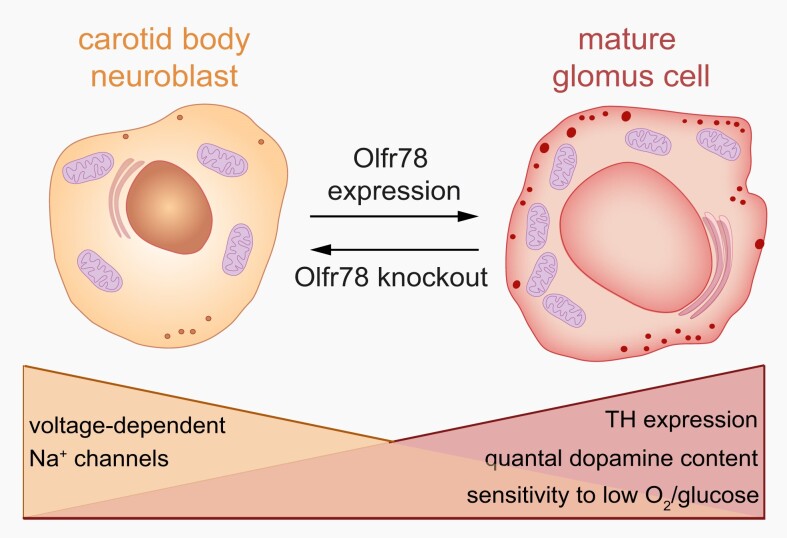

## Introduction

Olfactory receptor 78 (Olfr78) is a G-protein-coupled odorant receptor that is atypically expressed outside the olfactory epithelium. Olfr78 is expressed at high levels in the carotid body (CB),^1–3^ the prototypical acute oxygen (O_2_) sensor, and main organ responsible for the hypoxic ventilatory response (HVR).^4,5^ One group reported that Olfr78 acts as a lactate receptor in mice and that the HVR in constitutive Olfr78 knockout mice (Olfr78 −/− mice) is abolished and the CB responses to hypoxia blunted.^1^ Based on their findings, this group proposed a model whereby an increase in lactatemia during hypoxia and the subsequent endocrine activation of Olfr78-expressing CB glomus cells are responsible for the O_2_ regulation of breathing.^1^ This model has been challenged by several groups showing that lactate is a poor agonist for Olfr78,^2,6^ that Olfr78 is not a physiologically relevant lactate receptor,^7,8^ and that the HVR is not significantly altered in several strains of *Olfr78* −/− mice.^7^ It is well established that O_2_ sensing is an intrinsic property of CB glomus (or type I) cells^9–11^ and that these polymodal chemoreceptors are activated by low O_2_ tension and other stimuli, including lactate, via mechanisms that are unrelated to Olfr78.^4,12^ Nonetheless, one study reported that Olfr78 −/− mice have impaired HVR and glomus cell responses to mild hypoxia^8^ and a reduction in sympathetic activation and systemic hypertension induced by chronic intermittent hypoxia.^13^

*Olfr78* is one of the most abundant mRNA species expressed in CB glomus cells, but its functional significance remains poorly understood. As is the case for other genes relevant for CB glomus cell function,^14^  *Olfr78* mRNA levels also depend on the constitutively high expression of hypoxia-inducible factor 2 alpha (HIF2α).^15^ Therefore, it is conceivable that, although not essential for O_2_ sensing, Olfr78 has a role in glomus cell homeostasis. Importantly, all previous reports were about mice with a constitutive (or global) knockout of the *Olfr78* gene^16^ and therefore the phenotypes observed could result from pleiotropic effects on the various CB cell types and/or on various organs.^17^ In addition, the properties of Olfr78-deficient CB glomus cells have not been analyzed in detail.

Here, we report the generation and characterization of a C57BL/6-inbred conditional *Olfr78* knockout mouse strain, which we crossed with a gene-targeted TH-IRES-Cre driver strain in order to restrict the knockout of the gene to CB glomus cells and other catecholaminergic cell types. Offspring of this cross are henceforward referred to as TH-Olfr78 KO mice. We did not observe significant changes in the HVR of TH-Olfr78 KO mice. However, they exhibited a subtle but clearly reproducible glomus cell phenotype, with changes in mRNA expression, electrophysiological features, intracellular Ca^2+^ dynamics, and secretory activity that suggest alterations in the trophic maintenance of these cells. We speculate that Olfr78 contributes to maintaining a high basal activity of the adenyl cyclase-cyclic AMP pathway, which may be required for phenotypic specification of mature glomus cells.

## Materials and Methods

### Ethical Approval

In Frankfurt, mouse experiments were carried out in accordance with guidelines of the German Animal Welfare Act, the Directive 2010/63/EU of the European Parliament and of the Council, and the institutional ethical and animal welfare guidelines of the Max Planck Research Unit for Neurogenetics. Approval came from the *Regierungspräsidium* Darmstadt (Germany), and the *Veterinäramt* of the City of Frankfurt (Germany). In Seville, the Institutional Committee for Animal Care and Use at the University of Seville (21/04/2020/053 and 27/01/2017/018) approved all procedures used in this study. Handling of the animals was conducted in accordance with the European Community Council directive of September 22, 2010 (Directive 2010/63/EU) and the implementations of June 5, 2019 (Regulation 2019/2010) for the care and use of laboratory animals. To minimize the number of animals used in the experiments, mice subjected to in vivo analyses were subsequently euthanized and used for the in vitro studies. As all measurements of physiological constants done on mice were painless, analgesia was not used. For in vitro experiments, mice were killed by intraperitoneal injection with a lethal dose of anaesthetic (sodium thiopental, 120-200 mg/kg)

### Mouse Models

#### Generation of Tyrosine Hydroxylase Conditional Olfr78 Knockout Mice

The coding region of exon 4 of the *Olfr78* gene was flanked by *loxP* sites by gene targeting in the C57BL/6-derived embryonic stem cell line Bruce4. After obtaining germline transmission, the *neo*-selectable marker flanked by *FRT* sites was removed from the targeted mutation by crossing with Oz_flp, a C57BL/6-inbred strain that carries a knockin of the site-specific recombinase *flp* in the *ROSA26* locus driven by the *UbiC* promoter; the Oz_flp allele was crossed out subsequently. The strain carrying the floxed *Olfr78* allele without the Oz flip allele is publicly available from The Jackson Laboratory as stock #32801 with official strain name B6.Cg-Or51e2<tm3.1Mom>. Mice carrying the conditional Olfr78 knockout allele in the heterozygous state (Olfr78 f/+) or homozygous state (Olfr78 f/f) were crossed with TH-IRES-Cre mice.^18^ In this Cre driver strain, the gene-targeted *Th-IRES-Cre* knockin mutation provides Cre expression without disturbing expression of the tyrosine hydroxylase (*Th*) gene and affords efficient in vivo Cre-mediated recombination in catecholaminergic cells.^19,20^

#### Generation of Conditional HIF2α Knockout Mice

The *Epas1* (encoding HIF2α) conditional knock-out mice (EPAS1 KO: *Epas1* f/−, Cre) were generated by crossing mice carrying an *Epas1* floxed allele (The Jackson Laboratory, stock #008407)^21^ with mice carrying a tamoxifen-inducible Cre recombinase transgene.^22^ To induce recombination 2-mo-old mice were fed with tamoxifen-containing diet (TAM400/CreER; tamoxifen citrate, 400 mg/kg; Envigo) for a month. Thereafter, mice were kept on a normal diet for another 1.5 to 2 mo before experiments.

### Animal Care and Exposure of Mice to Chronic Hypoxia

Mice were housed at 22°C ± 1°C in a 12-h light/12-h dark cycle with ad libitum access to food (Teklad global 14% protein, Envigo) and water. Both male and female mice were used. In TH-Olfr78 KO mice maintained under chronic hypoxia (10% O_2_ atmosphere for 14 d), the experiments were performed using a hermetic chamber with control of O_2_, CO_2_, humidity, and temperature (Coy Laboratory Products). For the experiments, mice (2-3 mo-old) were maintained in standard rodent cages placed into the hypoxia chamber, with ad libitum access to pellet food and water, and within a 12‐h light/12-h dark cycle.

### Plethysmography

Plethysmography was used to study respiratory function in conscious unrestricted mice as previously described.^23^ Briefly, mice were placed in plethysmographic chambers (EMKA Technologies) and perfused at a constant flow rate (1 L/min) with air (21% O_2_, normoxia), and various gas mixtures: 10% O_2_, 12% O_2_ (hypoxia, maintained for 5 min once O_2_ percentage reached 10% or 12%), or 5% CO_2_ (hypercapnia, maintained during 1 min when CO_2_ percentage reached 5%). The hermetic chambers were provided with O_2_ and CO_2_ sensors to monitor the gas composition in parallel with changes in respiratory frequency recorded by a pressure sensor during the experiment. Data acquisition was performed using Iox2 (RRID:SCR_022973; EMKA Technologies). To calculate changes in respiratory frequency, the basal, hypoxic, and hypercapnic respiratory frequencies were estimated in each animal. Basal respiratory frequency was calculated by averaging the values acquired during the 160 s previous to exposure to hypoxia. Peak respiratory frequency (indicated by “*p*” in the figures) during hypoxia was calculated by averaging the values obtained during 110 s at the peak of the hypoxic response. The total average respiratory frequency (indicated by “*a*” in the figures) during exposure to hypoxia (10% or 12% O_2_) was estimated by the mean of values measured during the last 400 s of exposure to stimuli. Respiratory frequency during exposure to hypercapnia was estimated by averaging the values obtained during 90 s after reaching 4%-5% CO_2_ in the chamber before returning to normoxia.

### Hematocrit

Hematocrit was measured in blood samples collected from the carotid artery using a hematocrit tube located near the incision site (where the bifurcation was dissected) to allow blood to fill the tube by capillary action. Following blood collection, tubes were sealed with bone wax, placed in a microhematocrit centrifuge, and spun for 5 min at 3 g . Hematocrit was calculated manually by measuring the length of the column of packed red cells and total blood length and expressed as the percentage of erythrocytes relative to total blood volume (100%).

### Immunohistochemistry and Estimation of CB Volume

For immunohistochemical studies, mice were first perfused with phosphate buffered saline (PBS) and then with 4% paraformaldehyde in PBS before tissue dissection. Carotid bifurcations were fixed with 4% paraformaldehyde in PBS for 2 h, cryoprotected overnight with 30% sucrose in PBS, and embedded in O.C.T. (Cat# 4583, Tissue-Tek). Tissue sections of 10 μm were cut with a cryostat (Leica, Wetzlar) and incubated overnight at 4°C with primary antibodies: TH (1:100 dilution, Cat#AB1542, Sigma-Aldrich) and Ki67 (1:100 dilution, Cat#RM-9106, Epredia). This was followed by incubation with fluorescent secondary antibodies: Alexa Fluor 488 or Alexa Fluor 568 (1:500 or 1:1000 dilution, Cat#A11008 and Cat#A11004, Invitrogen, RRID: AB_143165 and RRID: AB_2534072). Nuclei were labeled with 4′,6′-diamidino-2-phenylindole (DAPI). Immunofluorescence images were obtained using a Nikon A1R+ confocal microscope. To estimate CB volume, serial sections of CBs were immunostained and the area within each CB section was calculated using Image J software (RRID:SCR_003070). Carotid body volume was calculated considering CB area in each section, section thickness, and total number of sections per CB.

### Carotid Body and Superior Cervical Ganglion Resection and Molecular Analyses

Mice were sacrificed by intraperitoneal administration of a lethal dose of sodium thiopental (120-150 mg/kg). Carotid bifurcations were removed and placed in cold PBS. Carotid bodies and superior cervical ganglion (SCG) were dissected. For real-time quantitative polymerase chain reaction (PCR) analysis, total RNA was isolated from CB and SCG using RNeasy Micro kit (Qiagen). For the analysis of CB gene expression profile in TH-Olfr78 KO and wild-type (WT) mice, each CB replicate was pooled from 3 mice to obtain enough amount of mRNA. Complementary RNA (cRNA) was then amplified from CB total RNA using the GeneChip WT PLUS Reagent Kit (Affymetrix). Total RNA (500 ng; or amplified cRNA in the case of CB) was reverse-transcribed into complementary DNA (cDNA) using the QuantiTect Reverse Transcription Kit (Cat#205311, Qiagen). Real-time quantitative PCR was performed on a 7500 Fast Real Time PCR System (Applied Biosystem). PCR was performed in duplicate in a total volume of 20 μL containing 1 μL of cDNA solution and 1 μL of TaqMan probe of the specific genes (Thermo Fisher Scientific). *Gapdh* mRNA was measured in each sample to normalize the amount of total RNA (or cRNA) input to perform relative quantifications.

### Enzymatic Dispersion of CB Cells and Preparation of CB Slices

Dispersed CB glomus cells were prepared as described previously.^24^ Briefly, dissected CBs were incubated for 20 min at 37°C in enzymatic solution [PBS pH 7.4 supplemented with 50 μm CaCl_2_, 0.6 mg/mL collagenase II (Cat#C6885, Sigma), 0.27 mg/mL trypsin (Cat#T8003, Sigma), and 1.25 U/mL porcine elastase (Cat#324682, Millipore)]. Then, CBs were mechanically stretched with needles and incubated for another 5 min at 37°C. After digestion, cells were mechanically dispersed by pipetting and centrifuged for 5 min at 300 *g*. The cell pellet was resuspended in culture medium DMEM (0 glucose)/DMEM-F-12 (Cat#11966-025/21331-020, Gibco) medium (3:1) supplemented with 100 U/mL penicillin and 10 mg/mL streptomycin (Cat#15140-122, Gibco), 2 m m l-glutamine (Cat#25030-024, Gibco), 10% fetal bovine serum (Cat#10270-106 Gibco), 84 U/L insulin (Actrapid, Cat#EU/1/02/230/011, Novonordisk), and plated on glass cover slips treated with poly-l-lysine (Cat#P1524, Sigma). Dispersed cells were maintained at 37°C in a 5% CO_2_ and 21% O_2_ incubator and used after 24 h of incubation.

To prepare CB slices, dissected CBs were placed in ice-cooled modified Tyrode solution and processed as described previously.^25,26^ Briefly, CB were included at 42ºC in 1% low melting point agarose (prepared in PBS). Agarose block containing CBs were glued to the platform a vibratome (VT1000S, Leica) to prepare sections of 150 μm. Carotid body sections were digested in enzymatic solution (the same used for dispersed glomus cells), during 5 min, in a water bath at 37ºC with shaking. Finally, slices were washed with PBS and incubated in the same culture medium used for dispersed glomus cells to which 1.2 U/mL of erythropoietin (Cat#EU 1/07/410/028, Sandoz) were added. Slices were incubated at 37°C in 5% CO_2_ for 24 h before use.

### Amperometry

To monitor single-cell secretory activity, dopamine secretion from glomus cells in CB slices was measured by amperometry.^23^ Secretory events were recorded with a 10 μm carbon fiber electrode polarized to +750 mV (to favor dopamine oxidation) using an external voltameter connected to the EPC-7 amplifier. Amperometric currents were recorded with an EPC-7 patch-clamp amplifier (HEKA Electronics, Lambrecht/Pfaltz). The signal was filtered at 100 Hz and digitized at 250 Hz before storage on computer. Data acquisition and analysis were carried out with an ITC-16 interface (Instrutech Corporation) and PULSE/PULSEFIT software (HEKA Electronics). For the experiments, a slice was transferred to the recording chamber of an upright microscope (Axioscope, Zeiss) and continuously perfused with extracellular solution (see the “Recording Solutions” section). The secretion rate (given in fC/min or pC/min) was calculated as the amount of charge transferred to the recording electrode during a given period of time. Experiments were performed at ∼35°C

### Microfluorimetric Measurements of Intracellular Ca^2+^

Microfluorimetric measurement of intracellular changes in Ca^2+^ concentration was performed in single dispersed glomus cells as previously described.^11,19^ Dispersed CB cells were loaded with Fura2-AM (TefLabsMW1002, Molecular Probes), 4 m m in DMEM/F-12 without serum at 37°C for 30 min and subsequently incubated for 15 min in complete medium to remove excess Fura2-AM. To perform the experiments, a coverslip with Fura 2-AM loaded cells was placed on a recording chamber mounted on the stage of a microscope equipped with epifluorescence and photometry. This set up consists of an inverted microscope (Nikon Eclipse Ti) equipped with a 40x/0.60 NA objective, a monochromator (Polychrome V, Till Photonics), and a CCD camera, controlled by Aquacosmos software (version 2.6, Hamamatsu Photonics). Alternating excitation wavelengths of 340 and 380 nm with emission wavelength of 510 nm were used to obtain the F340/F380 ratio.^11^ Background fluorescence was subtracted before obtaining the F340/F380 ratio. A dichroic FF409-Di03 (Semrock) and a band-pass filter FF01-510/84 (Semrock) were used. Cytosolic Ca^2+^ signals were digitized at a sampling interval of 500 ms. Experiments were performed at ∼35°C.

### Patch Clamp Recording and Electrophysiological Analyses

Macroscopic ionic currents were recorded from dispersed mouse glomus cells using the whole cell configuration of the patch clamp technique as adapted in our laboratory.^24,27^ Patch clamp electrodes (2-3 MΩ) were pulled from capillary glass tubes (Kimax, Kimble Products) with a horizontal pipette puller (Sutter instruments model *P*-1000) and fire-polished with a microforge (MF-830, Narishige). Voltage-clamp recordings were obtained with an EPC-7 patch clamp amplifier (HEKA Electronik) using standard voltage-clamp protocols designed with PULSE/PULSEFIT software (HEKA Electronik). The signal was filtered (10 kHz), subsequently digitized with an analog/digital converter (ITC-16 Instrutech Corporation), and finally sent to a computer. Data acquisition and storage were performed using the PULSE/PULSEFIT software (HEKA, Electronics) at a sampling interval of 20 μs. Experiments were performed at ∼35°C.

### Recording Solutions

Dispersed cells or slices used for in vitro amperometric or microfluorimetric recordings were transferred to the recording chamber and continuously perfused with a control solution containing 125 m m NaCl, 4.5 m m KCl, 23 m m NaHCO_3_, 1 m m MgCl_2_, 2.5 m m CaCl_2_, 5 m m glucose, and 5 m m sucrose at ∼35°C. In 40 m m K^+^ solution, NaCl was replaced equimolarly with KCl. The “normoxic” solution was bubbled with a gas mixture of 20% O_2_, 5% CO_2_, and 75% N_2_ (O_2_ tension ∼145 mmHg). The “hypoxic” solution was bubbled with 5% CO_2_ and 95% N_2_ to reach an O_2_ tension of ∼10 to 15 mmHg in the chamber. Osmolality of solutions was ∼300 mosm/Kg with pH 7.4. The hypercapnic solution was bubbled with a gas mixture of 20% O_2_, 20% CO_2_. The low glucose experiments were done upon exposure of the cells to solutions in which sucrose replaced glucose (125 mM NaCl, 4.5 mM KCl, 23 mM NaHCO_3_, 1 mM MgCl_2_, 2.5 mM CaCl_2_, 0 mM glucose, and 10 mM sucrose or 0.5 mM glucose and 9.5 mM sucrose).

To monitor macroscopic Ca^2+^, Na^+^, and K^+^ currents, glomus cells were perfused with external solutions and dialyzed with internal solutions. Solutions used to record whole-cell currents through Na^+^ and Ca^2+^ channels (using Ba^2+^ as a charge carrier) contained the following: external solution: 140 m m NaCl,10 m m BaCl_2_, 4.7 m m KCl, 10 m m Hepes, and 10 m m glucose (pH 7.4) (osmolality, 300 mOsm/kg); internal solution: 130 m m CsCl, 10 m m EGTA, 10 m m Hepes, and 4 m m adenosine triphosphate (ATP-Mg) (pH 7.2; osmolality, 285 mOsm/kg). In some experiments, a 0 Na^+^ solution was used, in which external Na^+^ was completely replaced with the impermeant cation N-methyl D-glucamine. The external solution used to record whole-cell K^+^ currents was the same control solution used for amperometric and microflourimetric analysis, and the internal solution contained 80 m m K^+^ glutamate, 50 m m KCl, 1 mm MgCl_2_, 10 m m Hepes, 4 m m ATP-Mg, and 5 m m EGTA (pH 7.2). In the high K^+^ solutions, KCl replaced NaCl equimolarly. In experiments where short chain lipid compounds were used, they also replaced NaCl equimolarly at the concentrations indicated.

### Statistical Analysis

Statistical analysis was carried out using Prism Version 8.2.1. (279) for MacOS. Normality of the data sets obtained in the experiments was tested with the Shapiro-Wilk, D'Agostino & Pearson, or Kolmogorov-Smirnov test. In some cases, a log transformation was performed to normalize the distribution prior to parametric analysis. All tests, parametric and nonparametric, were two-tailed. A *P* < 0.05 was considered statistically significant. Data with normal distribution are described as mean, and standard error of the mean with the number (*n*) of experiments indicated. For graphical representation of data, bar diagrams are used, with indication of the mean ± SEM, and scatter plots of the data points superimposed. For graphical representation of data that do not follow a normal distribution, box plots are used with indication of median, quartiles, and outliers. To facilitate comparisons, mean and SEM values are also given in the figure legends.

## Results

### Expression of Genes Relevant to CB Function in TH-Olfr78 Knockout Mice

**Figure 1.**
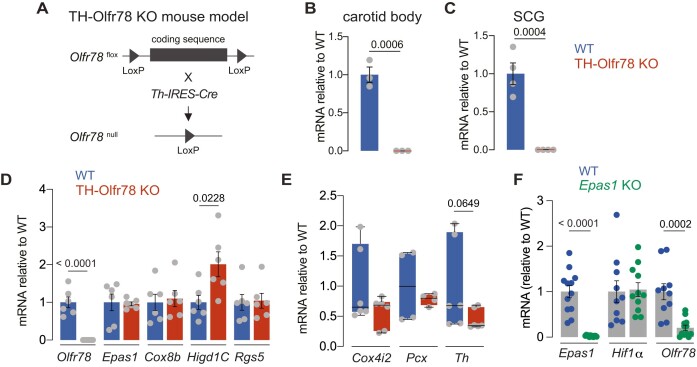
Expression of genes relevant to carotid body (CB) function in TH-Olfr78 KO and *Epas1* KO mice. (**A**) Scheme of the generation of the TH-Olfr78 KO mouse model. (**B**) and (**C**) Levels of *Olfr78* mRNA, relative to wild type (WT), in CB samples (**B**) and in superior cervical ganglion (SCG) samples (**C**) of WT mice and TH-Olfr78 KO mice. Data are expressed as mean ± SEM with individual values superimposed. Values are CB (WT: 1 ± 0.10; KO: 0.00046 ± 0.00008, *n* = 3 replicates/group); SCG (WT: 1 ± 0.14; KO: 0.0009 ± 0.0001, *n* = 4 replicates/group). Statistically significant *P*-values (<0.05) calculated by unpaired two-tailed *t*-test are indicated. (**D**) and (**E**) Levels of mRNA, relative to WT, of genes relevant to glomus cell function: *Olfr78, Epas1, Cox8b, Higd1C*, and *Rgs5* (**D**) and *Cox4i2, Pcx, and Th* (**E**) in CB samples of WT mice and TH-Olfr78 KO mice. Data with normal distribution are presented by bar diagrams (**D**) and expressed as mean ± SEM with data values superimposed. Values that do not follow a normal distribution are represented as boxplots (**E**) indicating median (middle line), 25th, 75th percentile (box), and largest and smallest values (whiskers). All data are plotted individually. *P*-values (<0.05 or near this value) calculated with unpaired two-tailed *t*-test (**D**) and with Mann-Whitney test (**E**) are indicated. Mean ± SEM values in panel **D**: *Olfr78* (WT: 1 ± 0.14; KO; 0.0004 ± 0.00005); *Epas1* (WT: 1 ± 0.22; KO: 0.96 ± 0.05), *Cox8b* (WT: 1 ± 0.21; KO: 1.1 ± 0.21), *Higd1C* (WT: 1 ± 0.18; KO: 2 ± 0.33), and *Rgs5* (WT: 1 ± 0.21; KO: 1 ± 0.20); *n* = 6 replicates/group. Mean ± SEM values in panel **E** are *Cox4i2* (WT: 1 ± 0.26; KO: 0.57 ± 0.11), *Pcx* (WT: 1 ± 0.31; KO: 0.78 ± 0.48), and *Th* (WT: 1 ± 0.30; KO: 0.45 ± 0.07); *n* = 6 replicates/group. (**F**) Levels of mRNA of *Epas1, Hif1α*, and *Olfr78* in CB samples of conditional *Epas1* KO mice relative to WT mice. Data are expressed as mean ± SEM with all data values superimposed. Values are *Epas1* (WT: 1 ± 0.14; KO: 0.03 ± 0.005), *Hif1α* (WT: 1 ± 0.24; KO: 1.05 ± 0.015), and *Olfr78* (WT: 1 ± 0.18 and KO, 0.21 ± 0.06); *n* = 10-12 replicates/group. *P*-values (<0.05) calculated by unpaired two-tailed *t*-test are indicated.

Mice carrying the newly generated floxed *Olfr78* allele were crossed with gene-targeted knockin mice expressing Cre recombinase under the control of the tyrosine hydroxylase (*Th*) promoter.^18,19^ The resulting mice (TH-Olfr78 KO mice; Figure 1A) showed a complete absence of *Olfr78* mRNA in CB glomus cells (Figure 1B) and other catecholaminergic tissues such as the SCG (Figure 1C). Next, we studied differences in the mRNA expression levels of a set of genes that are known to be relevant for CB glomus cell function.^2,3,14,28,29^ These included *Epas1* (encoding HIF2α), 3 atypical mitochondrial complex IV (MCIV) subunit isoforms (*Cox4i2, Cox8b*, and *Higd1C*), pyruvate carboxylase (*Pcx*), regulator of G-protein-signaling 5 (*Rgs5*), and tyrosine hydroxylase (*Th*), which is expressed in the highly dopaminergic mature glomus cells. Among these genes, *Higd1C*, which has been suggested to contribute to the characteristically high sensitivity of glomus cell mitochondria to hypoxia,^28^ was significantly upregulated in Olfr78-deficient cells (Figure 1D and E). There was also a trend for *Th* mRNA to be downregulated in TH-Olfr78 KO mice but the data do not reach statistical significance (Figure 1E). As is the case for other genes critically involved in adult CB function,^14,15^  *Olfr78* mRNA expression appears to be dependent on HIF2*α* (Figure 1F).

### In Vivo Responses to Acute and Chronic Hypoxia

The CB is the prototypical acute O_2_-sensing organ in adult mammals and main organ that is responsible for the HVR.^4,5^ To evaluate the responsiveness of WT mice and TH-Olfr78 KO mice to hypoxia, we tested the acute effects of O_2_/CO_2_ on ventilation and the changes in CB structure induced by sustained hypoxia.

**Figure 2.**
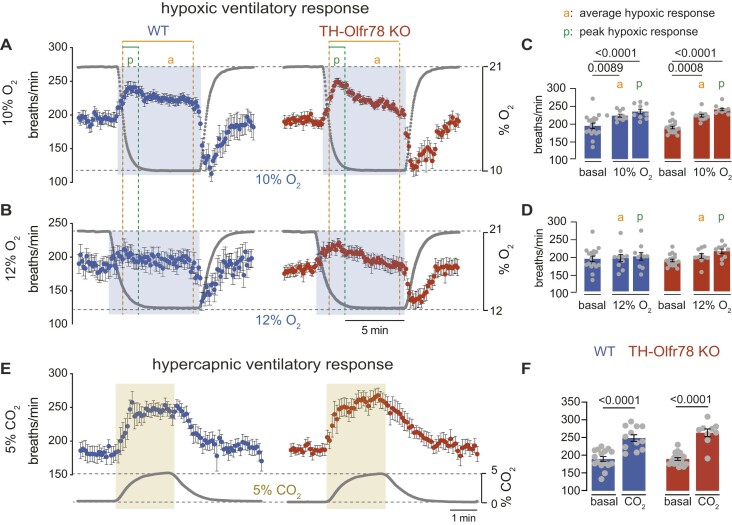
Hypoxic and hypercapnic ventilatory responses in wild-type (WT) mice and TH-Olfr78 KO mice. (**A**), (**B**), and (**E**) Time course of the increase in respiratory frequency (breaths/min, left ordinate axis) induced by hypoxia (10% O_2_, **A** and 12% O_2_, **B**) and hypercapnia (5% CO_2_, **E**) in WT mice (10% O_2_, *n* = 10, 12% O_2_, *n* = 10, and 5% CO_2_, *n* = 10) and TH-Olfr78 KO mice (10% O_2_, *n* = 11, 12% O_2_, *n* = 10, and 5% CO_2_, *n* = 11). Changes in % O_2_ and % CO_2_ over time are presented related to the right ordinate axis. (**C**), (**D**), and (**F**) Respiratory frequency (in breaths/min) during normoxia (basal, 21% O_2_), hypoxia (10% O_2_, **C** and 12% O_2_, **D**) and hypercapnia (20% CO_2_, **F**) measured by plethysmography in WT mice and TH-Olfr78 KO mice. Respiratory frequency during hypoxia was calculated at the peak (box and “*p*” indicated in panels **A** and **B**) and during the whole stimuli (box and “*a*” in panel **A** and **B**). Data points are presented as bar diagrams indicating mean ± SEM with all data points superimposed. Values (in breaths/min) are WT mice (basal: WT, 194.2 ± 8; KO, 189.3 ± 4; average 10% O_2_: WT, 223.4 ± 4; KO, 223.2 ± 4; peak 10% O_2_: WT, 235.3 ± 7; KO, 241.1 ± 3; average 12% O_2_: WT, 196.2 ± 10; KO, 202.3 ± 7; peak 12% O_2_: WT, 201.3 ± 11; KO, 216.0 ± 6; and CO_2_: WT, 248.1 ± 9; KO, 262.7 ± 11). Statistically significant *P*-values calculated by one-way ANOVA followed by Tukey’s multiple comparisons test are indicated.

Mice exposed acutely to either 10% or 12% O_2_ responded with rapid graded increases in respiration rate that have a peak (lasting for ∼1 min) at the onset of the response, followed by a progressive decline due to the inhibitory effect of hypocapnia caused by hyperventilation (Figure 2A-D). Neither the peak (“*p*” in the figure) nor the average (“*a*” in the figure) increases in breathing frequency induced by lowering environmental O_2_ tension from normal air (21% O_2_) to either 10% O_2_ or 12% O_2_ are different between WT mice and TH-Olfr78 KO mice (Figure 2A-D). Exposure to hypercapnia (5% CO_2_ in a 21% O_2_ atmosphere) produced a robust and sustained increase in breathing frequency, which is also similar in the 2 mouse models (Figure 2E and F). These data confirm our findings in mice with a constitutive (or global) knockout of the *Olfr78* gene^7^ and indicate that Olfr78 deficiency does not alter O_2_/CO_2_ regulation of breathing within the range of O_2_ tensions that are normally used to monitor the HVR.

**Figure 3.**
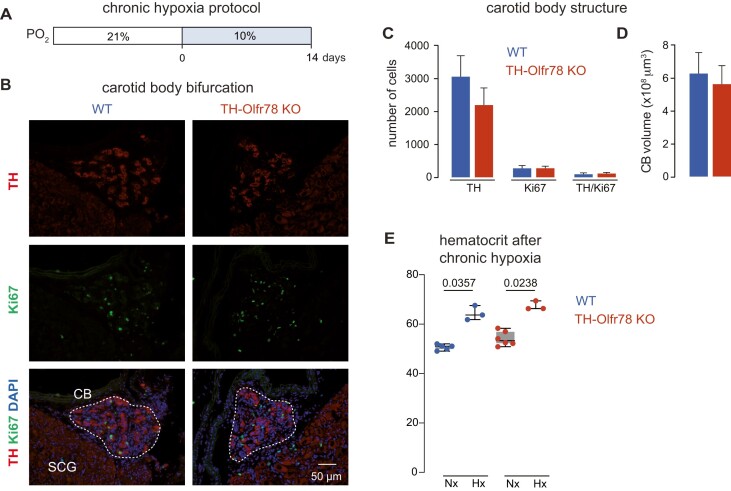
Changes in carotid body (CB) structure upon chronic hypoxia. (**A**) Scheme of the chronic hypoxia protocol. (**B**) Immunohistochemical analyses of carotid bifurcations in wild-type (WT) mice (left) and TH-Olfr78 KO mice (right) exposed to chronic hypoxia (14 d, 10% O_2_). The typical growth of the TH + glomus cell mass under hypoxia (upper panel) is also observed in TH-Olfr78 KO mice. Proliferating cells were labeled with Ki67 (middle panel). Colocalization of TH and Ki67 is represented in the lower panel, where nuclei were labeled with DAPI. CB, carotid body; SCG, superior cervical ganglion. (**C**) Average number of TH + cells, Ki67 + cells, and double TH/Ki67 + cells in CB of WT mice and TH-Olfr78 KO mice. Data are expressed as mean ± SEM. Values are TH+ (WT, 3062 ± 626, *n* = 3; KO, 2200 ± 511, *n* = 3), Ki67+ (WT, 282 ± 75, *n* = 3; KO, 284 ± 54, *n* = 3), and double TH/Ki67+ (WT, 102 ± 35, *n* = 3; KO, 124 ± 35, *n* = 3). (**D**) Quantification of CB volume of WT mice and TH-Olfr78 KO mice. Data (in μm^3^) are presented as mean ± SEM. WT, 6.3 × 10^8^ ± 1.2 × 10^8^; KO, 5.6 × 10^8^ ± 1.1 × 10^8^). (**E**) Average hematocrit values measured in WT mice and TH-Olfr78 KO mice in normoxia (Nx) and after exposure to chronic hypoxia (Hx). Data are presented as boxplots indicating median (middle line), 25th, 75th percentile (box), and largest and smallest values range (whiskers), and with all data values superimposed. Mean ± SEM are normoxia, WT, 50.7 ± 0.5 (*n* = 5); KO, 54.2 ± 1.2 (*n* = 6); chronic hypoxia, WT, 64.4 ± 1.7 (*n* = 3); KO, 67.4 ± 1 (*n* = 3). *P*-values (<0.05) calculated with Mann-Whitney *t*-test are indicated. The increase in hematocrit is similar in both groups of mice.

The CB grows severalfold in size upon exposure to chronic (days/weeks) hypoxia.^30^ This well-known adaptive response ensures the sustained activation of the respiratory center that is required for acclimatization to hypoxic environments. Carotid body growth upon chronic hypoxia requires the activation of CB glomus cells,^31^ which release transmitters that rapidly induce proliferation and maturation of neighboring TH-positive CB neuroblasts (immature glomus cells) followed by activation of pluripotent CB stem cells.^17,32^ We found that the number of TH-positive cells, proliferating cells (Ki67 positive), and the CB volume are comparable in WT mice and TH-Olfr78 KO mice maintained in a hypoxic environment (10% O_2_) for 2 wk (Figure 3A-D). Moreover, the increase in hematocrit after exposure to chronic hypoxia, a parameter that is augmented in mice with altered CB O_2_ sensing and no hyperventilation in response to hypoxia,^20^ is similar in WT mice and TH-Olfr78 KO mice (Figure 3E). Taken together, we did not detect any differences of note in the in vivo adaptive responses to sustained hypoxia between WT mice and TH-Olfr78 KO mice.

### Electrophysiology of Glomus Cells

**Figure 4.**
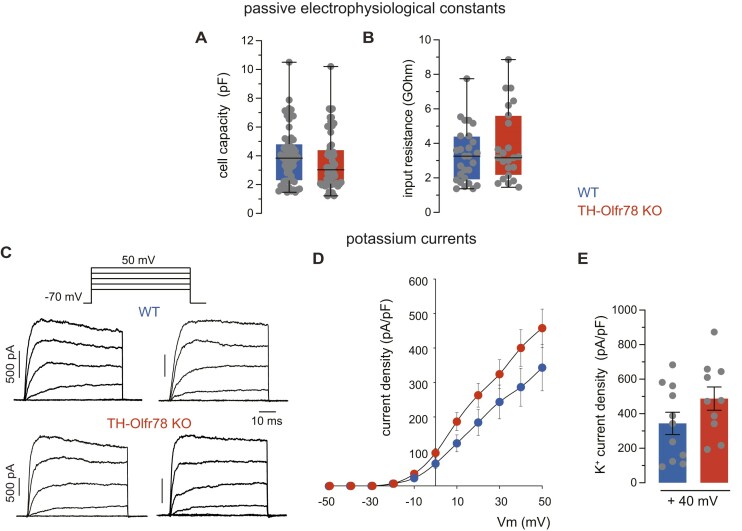
Passive electrical parameters and voltage-dependent K^+^ currents of dispersed glomus cells of wild-type (WT) mice and TH-Olfr78 KO mice. (**A**) and (**B**) Average cell capacitance (in pF, **A**) and input resistance (in GOhm, **B**) measured in glomus cells of WT mice and TH-Olfr78 KO mice. Data are presented as boxplots indicating median (middle line), 25th, 75th percentile (box), and largest and smallest values range (whiskers), and with all data values superimposed. Mean ± SEM are: capacitance, WT, 3.955 ± 0.245 (*n* = 61); KO, 3.589 ± 0.0.251 (*n* = 59); input resistance, WT, 3.298 ± 0.259 (*n* = 48); KO, 3.726 ± 0.293 (*n* = 44). (**C**) Top, representation of the depolarizing pulse protocol (50-ms depolarizing pulses reaching membrane potentials between −30 to +50 mV in steps of 20 mV from a holding potential of −70 mV). Families of representative macroscopic K^+^ currents recorded from dispersed glomus cells of WT mice (middle) and TH-Olfr78 KO mice (bottom) illustrating the variability in the time course of the current in both types of mice. (**D**) Average maximal K^+^ current density (in pA/pF, ordinate) versus voltage (in mV, abscissa) relationship in glomus cells of WT mice and TH-Olfr78 KO mice. Each point is the average of 11 (WT) and 10 (KO) different experiments. (**E**) Average maximal K^+^ current density (in pA/pF) recorded in response to depolarizing pulses to +40 mV in isolated glomus cells of WT mice and TH-Olfr78 KO mice. Data are expressed as mean ± SEM. Values are TH+ (WT, 344 ± 64, *n* = 11; KO, 487 ± 67, *n* = 10).

**Figure 5.**
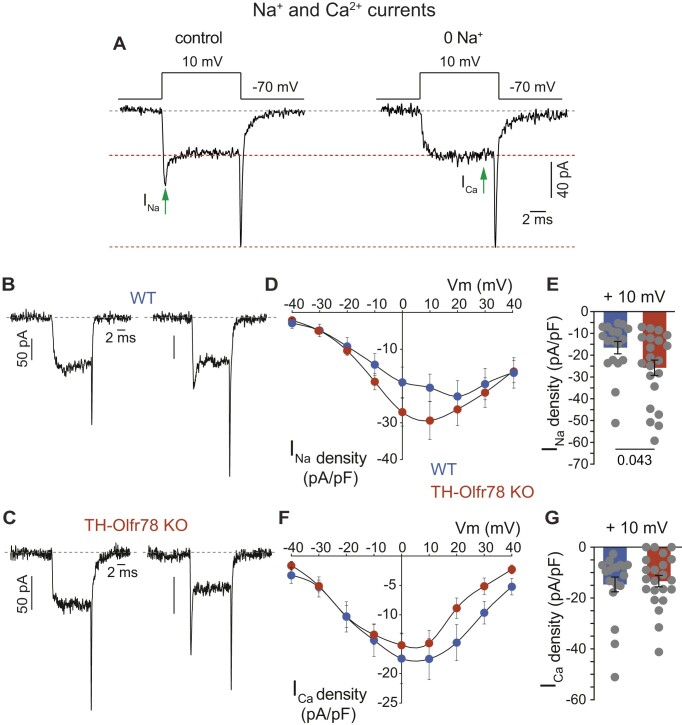
Voltage-dependent Na^+^ and Ca^2+^ currents of dispersed glomus cells of wild-type (WT) mice and TH-Olfr78 KO mice. (**A**) Macroscopic Na^+^ and Ca^2+^ currents elicited by membrane depolarization in a patch clamped glomus cell in the presence (left) and absence (right) of extracellular Na^+^. Complete replacement of Na^+^ was achieved with the impermeant cation N-methyl glucamine. Na^+^ current (I_Na_) and Ca^2+^ current (I_Ca_) were measured at the indicated time. (**B**) and (**C**) Representative examples of macroscopic inward currents with variable components of Na^+^ and Ca^2+^ current recorded from glomus cells of WT mice (**B**) and TH-Olfr78 KO mice (**C**). Standard (full Na^+^) external solution and pulse protocol are as illustrated in panel **A** (left). (**D**) and (**F**) Average Na^+^ (**D**) and Ca^2+^ (**F**) current density (in pA/pF, ordinate) versus voltage (in mV, abscissa) relationship in glomus cells of WT mice and TH-Olfr78 KO mice. Each point is the average of 18 (WT) and 21 (KO) different experiments. (**E**) and (**G**) Average Na^+^ (**E**) and Ca^2+^ (**G**) current density (in pA/pF) recorded in response to depolarizing pulses to +10 mV from glomus cells of WT mice and TH-Olfr78 KO mice. Values, expressed as mean ± SEM, are Na^+^ current (WT, −16.5 ± 2.8, *n* = 18; KO, −25.8 ± 3.6, *n* = 21); Ca^2+^ current (WT, −14.6 ± 2.9, *n* = 19; KO, −13.3 ± 2.2, *n* = 21). *P*-values (<0.05) calculated with unpaired two-tailed *t*-test are indicated in panels (**E**) and (**G**).

**Figure 6.**
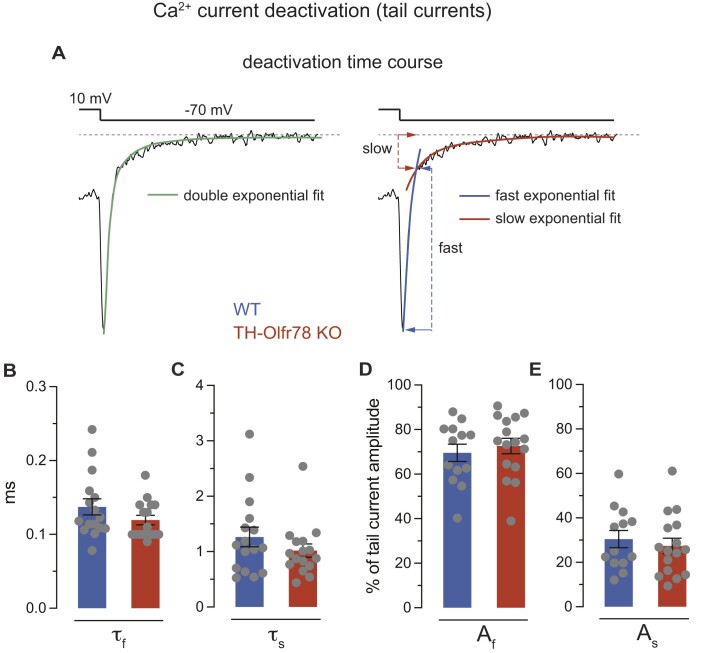
Components of Ca^2+^ currents of dispersed glomus cells of wild-type (WT) mice and TH-Olfr78 KO mice. (**A**) Representative Ca^2+^ tail current recorded from a glomus cell at the end of a 10-ms depolarizing pulse to +10 mV upon repolarization to −70 mV. Deactivation time course is fitted by a double exponential function (left) with fast and slow components (right). (**B**) and (**C**) Average deactivation time constants (in ms) for the fast (τ_f_, **B**) and slow (τ_s_, **C**) component of the tail Ca^2+^ currents recorded from glomus cells of WT mice and TH-Olfr78 KO mice. Pulse protocol as indicated in (**A**). Data are expressed as mean ± SEM. Values are τ_f_ (WT, 0.1373 ± 0.010, *n* = 16; KO, 0.1194 ± 0.006, *n* = 16); τ_s_ (WT, 1.264 ± 0.177, *n* = 16; KO, 1.017 ± 0.120, *n* = 16). (**D**) and (**E**) Average % of tail current amplitude for the fast (**D**) and slow (**E**) component of the tail Ca^2+^ current recorded from glomus cells of WT mice and TH-Olfr78 KO mice. Data are expressed as mean ± SEM. Values are A_f_ (WT, 0.1373 ± 0.010, *n* = 16; KO, 0.1194 ± 0.006, *n* = 16); A_s_ (WT, 69.55 ± 3.86, *n* = 13; KO, 72.6 ± 3.47, *n* = 16).

The electrical properties of dispersed glomus cells of WT mice and TH-Olfr78 KO mice were recorded with the whole-cell configuration of the patch clamp technique. Average passive electrical parameters (cell capacitance and input resistance) are similar in the two cell types, although there is a trend for cell capacitance (proportional to cell size) to be smaller in Olfr78-deficient cells (Figure 4A and B). All cells showed robust macroscopic voltage-dependent K^+^ currents. The time course of the K^+^ currents varied among glomus cells of WT and TH-Olfr78 KO mice, suggesting that different subtypes of voltage-gated K^+^ channels with variable activation and inactivation kinetics are expressed in these cells (Figure 4C). The peak K^+^ current amplitude (presented as current density) at membrane potentials >10 mV tends to be larger in Olfr78-deficient cells (Figure 4D and E). Voltage-dependent inward Na^+^ and Ca^2+^ currents were recorded in WT mice and TH-Olfr78 KO mice glomus cells dialyzed with Cs^+^ (to block K^+^ channels). Application of 10-ms depolarization pulses induces in all cells inward Ca^2+^ currents, which, in some cases, are preceded by a fast and transient current (Figure 5A-C). This transient component of the current is selectively abolished after replacement of external Na^+^ with the impermeant cation N-methyl glucamine (I_Na_, Figure 5A), suggesting that it is due to the opening of rapidly inactivating Na^+^ channels. The Ca^2+^ current, which is unaffected by Na^+^ removal, remains stable during the pulse (I_Ca_, Figure 5A). The number of cells expressing a measurable Na^+^ current is larger in TH-Olfr78 KO mice (82%, *n* = 22 cells) than in WT mice (54%, *n* = 24 cells). In addition, Olfr78-deficient cells have larger peak Na^+^ current density (I_Na_ at +10 mV) in comparison to WT cells (Figure 5D and E). Small differences in the density of Ca^2+^ channel currents (I_Ca_ at +10 mV) between Olfr78-deficient cells and WT cells are not statistically significant (Figure 5F and G). The time constant and amplitude of the fast and slow deactivating components of the tail currents (Figure 6A), representing the two main Ca^2+^ channel classes expressed in glomus cells,^33–35^ are not affected by Olfr78 deficiency (Figure 6B-E). Taken together, these data reveal clear differences in the level of Na^+^ channel expression between CB glomus cells of WT and TH-Olfr78 KO mice. Olfr78-deficient cells appeared to also display subtle differences in capacitance (smaller cell size) and in K^+^ and Ca^2+^ current density, but the results are not statistically significant.

### Neurosecretory Responses of Glomus Cells to Hypoxia and Other Stimuli

**Figure 7.**
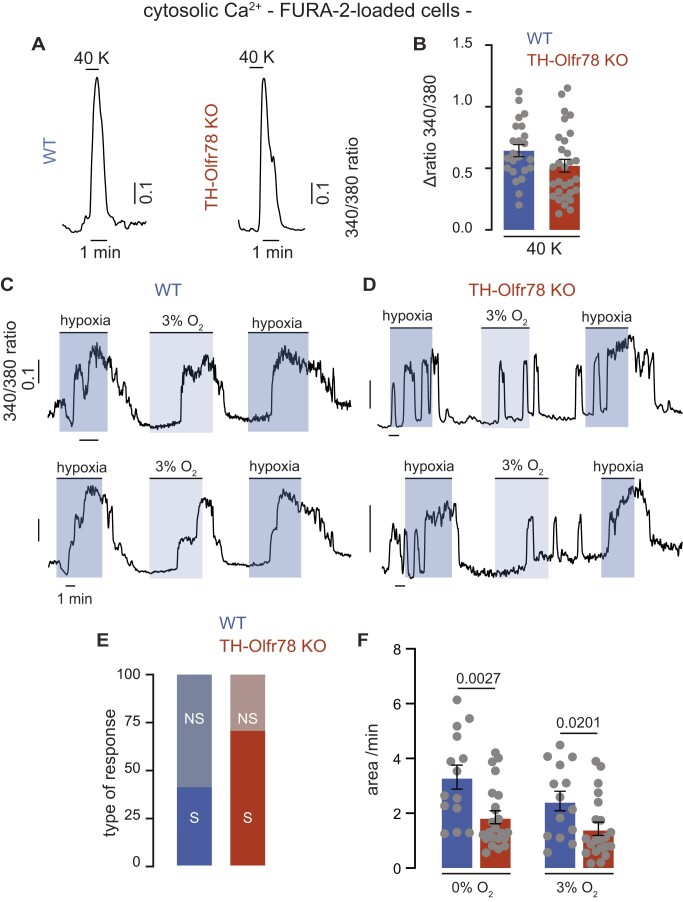
Changes in cytosolic [Ca^2+^] elicited by high extracellular K^+^ and hypoxia in glomus cells of wild-type (WT) mice and TH-Olfr78 KO mice. (**A**) Representative recordings of the changes in cytosolic Ca^2+^ (measured as the increase in the 340/360 fluorescence ratio) recorded in single Fura-2-loaded dispersed glomus cells from WT mice (left) and TH-Olfr78 KO mice (right) in response to depolarization with high KCl (40 m m). (**B**) Increase in cytosolic [Ca^2+^] elicited by 40 m m K^+^ in dispersed glomus cells of WT mice mice and TH-Olfr78 KO mice. Data are presented as mean ± SEM. Values (in fluorescence ratio) are (WT, 0.597 ± 0.047, *n* = 18; KO, 0.555 ± 0.058, *n* = 25). (**C**) and (**D**) Representative recordings of nonspiking (**C**) and spiking (**D**) cytosolic [Ca^2+^] signals recorded in dispersed FURA-2-loaded glomus cells of WT mice (**C**) and TH-Olfr78 KO mice (**D**) in response to different levels of hypoxia (external solutions bubbled with either 0% O_2_ ∼15 mmHg or 3% O_2_ ∼30 mmHg). (**E**) Percentage of spiking (S) and nonspiking (NS) hypoxia-induced Ca^2+^ responses recorded in WT mice (*n* = 44/6 cells/mice) and TH-Olfr78 KO mice (*n* = 41/5 cells/mice). (**F**) Quantification of the cytosolic Ca^2+^ changes (area under the curve in arbitrary units/min) induced by hypoxia in WT mice and TH-Olfr78 KO mice. Data are represented as mean ± SEM. Area under curve (a.u./min): WT: hypoxia (3.32 ± 0.44, *n* = 14/4 cells/mice); 3% O_2_ (2.45 ± 0.35, *n* = 14/4 cells/mice); TH-Olfr78 KO: hypoxia (1.85 ± 0.23, *n* = 23/3 cells/mice); 3% O_2_ (1.31 ± 0.24, *n* = 23/3 cells/mice). *P*-values were calculated with unpaired two tails *t*-test.

Glomus cells are O_2_-sensitive neurosecretory elements that respond to hypoxia with membrane depolarization, Ca^2+^ influx, and an increase in cytosolic [Ca^2+^]. This Ca^2+^ signal triggers the exocytotic release of transmitters that activate afferent sensory fibers impinging on brain respiratory centers.^30^ We monitored changes in cytosolic [Ca^2+^] by microfluorimetry in Fura-2-loaded dispersed glomus cells. Brief exposure to an externally applied depolarizing solution with high K^+^ induced smooth Ca^2+^ signals of large amplitude, which developed in parallel with the time course of bath solution exchange. The amplitude of these signals is similar in glomus cells of WT mice and TH-Olfr78 KO mice (Figure 7A and B). We also observed, both in glomus cells of WT mice and TH-Olfr78 KO mice, the presence of highly reversible hypoxia-induced Ca^2+^ signals that varied in amplitude and duration depending on the O_2_ tension in the bath solution (hypoxia, ∼15 mmHg; 3% O_2_, ∼30 mmHg) (see representative traces in Figure 7C and D). In some cases, the hypoxia-induced Ca^2+^ signal had an oscillatory pattern, which was more frequent in Olfr78-deficient glomus cells. Clear repetitive Ca^2+^ spiking signals were observed in more than 70% of cells of TH-Olfr78 KO mice but only in ∼40% of cells of WT mice (Figure 7E). Quantification of the area under the Ca^2+^ signals induced by hypoxia indicates that cells of TH-Olfr78 KO mice are less responsive to hypoxia than cells of WT mice (Figure 7F).

**Figure 8.**
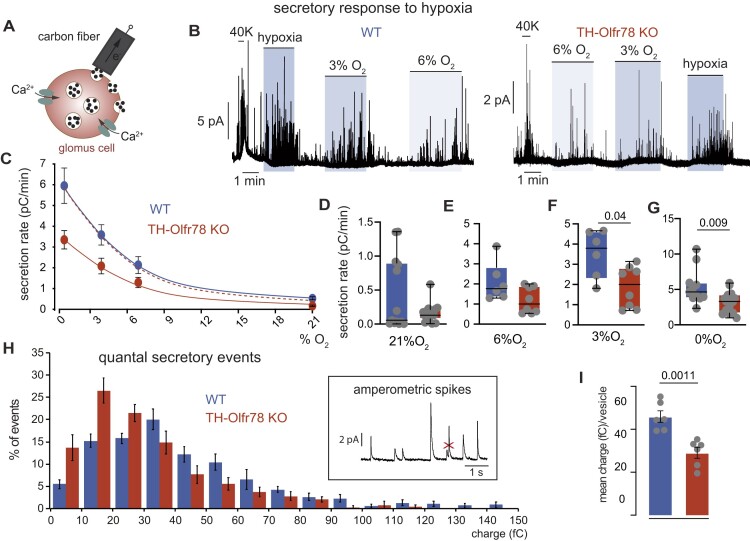
Secretory response to hypoxia of glomus cells in carotid body (CB) slices from wild-type (WT) mice and TH-Olfr78 KO mice. (**A**) Scheme illustrating the amperometric recording of quantal dopamine release from a single glomus cell (modified from Torres-Torrelo et al. 2021). (**B**) Representative amperometric recordings of the secretory activity recorded from glomus cells of WT mice (left) and TH-Olfr78 KO mice (right) under normoxia (21% O_2_, 150 mmHg) and during exposure to hypoxia (6% O_2_ ∼50 mmHg; 3% O_2_ ∼30 mmHg; 0% O_2_, ∼15 mmHg) and depolarization with potassium (40 m m K^+^). (**C**) Average secretion rate (ordinate) elicited by different levels of hypoxia (abscissa) in glomus cells of WT mice and TH-Olfr78 KO mice. Values in picocoulombs/min are (mean ± SEM): WT (21% O_2_, 0.43 ± 0.15, *n* = 13; 6% O_2_, 2.1 ± 0.4, *n* = 6; 3% O_2_, 3.52 ± 0.5, *n* = 6; 0% O_2_, 6 ± 0.7, *n* = 10); KO (21% O_2_, 0.2 ± 0.03, *n* = 13; 6% O_2_, 1.3 ± 0.24, *n* = 7; 3% O_2_, 2 ± 0.4, *n* = 7; 0% O_2_, 3.3 ± 0.4, *n* = 14). Lines have been drawn by eye. Discontinuous line was obtained by scaling the red line. (**D**-**G**) Boxplots representing average secretion rate in basal conditions 21% O_2_ (**D**), 6% O_2_ (**E**), 3% O_2_ (**F**), and 0% O_2_ (**G**). The boxplots indicate median (middle line), 25th, 75th percentile (box), and largest and smallest values range (whiskers). All data were plotted individually. *P*-values were calculated with Mann-Whitney test. *P*-values <0.05 are presented in panels (**F**) and (**G**). (**H**) Frequency-charge distribution of individual exocytotic events elicited in response to hypoxia from glomus cells in CB slices prepared from WT mice (*n* = 393 spikes, 6 mice) and TH-Olfr78 KO mice (*n* = 384 spikes, 6 mice). The inset shows examples of quantal secretory events. Compound secretory events, as the example indicated by a cross, were discarded for the quantification. (**I**) Mean vesicle charge obtained from glomus cells of WT mice and TH-Olfr78 KO mice. Values in femtocoulombs are (mean ± SEM). WT: 46 ± 3, *n* = 393 spikes; KO: 29 ± 3, *n* = 384 spikes. *P*-values were calculated with unpaired two tails *t*-test.

**Figure 9.**
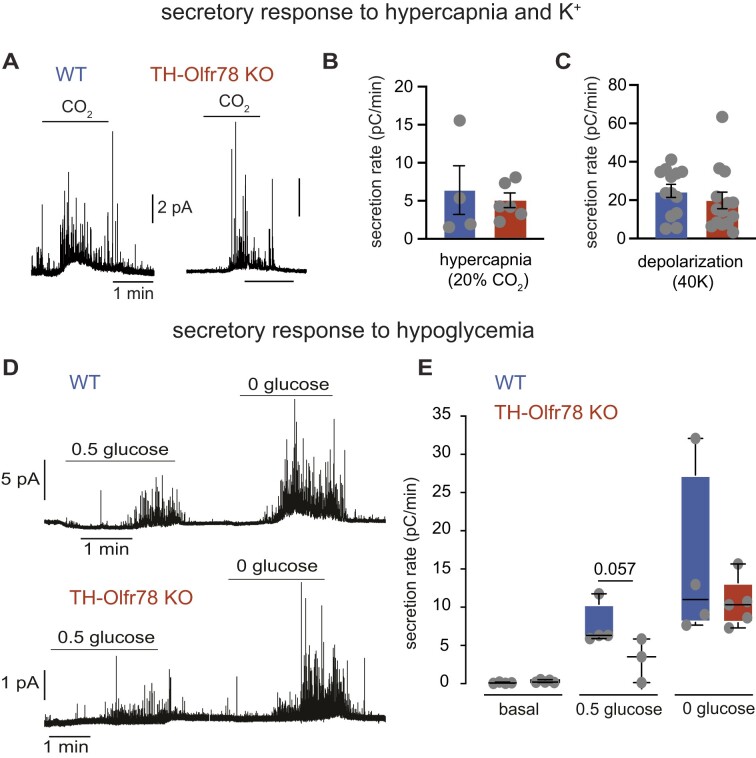
Secretory response to hypercapnia, K^+^, and hypoglycemia of glomus cells of wild-type (WT) mice and TH-Olfr78 KO mice. (**A**) Amperometric recordings of catecholamine release in response to hypercapnia (20% CO_2_) of glomus cells of WT mice (left) and TH-Olfr78 KO mice (right). (**B**) and (**C**) Average secretion rates (in pC/min) during exposure of glomus cells of WT mice and TH-Olfr78 KO mice to hypercapnia (20% CO_2_, **B**) and depolarization with 40 m m K^+^ (**C**). Data are presented as bar diagrams indicating mean ± SEM. Values are hypercapnia (WT: 6.09 ± 3.3 pC/min, *n* = 4 cells exp; KO, 4.91 ± 0.9, *n* = 6 cells); 40 m m K^+^ (WT: 24.43 ± 3.4 pC/min, *n* = 13 cells; KO, 19.15 ± 4.3, *n* = 14 cells). (**D**) Representative amperometric signal showing catecholamine release from glomus cells of WT mice (top) and TH-Olfr78 KO mice (bottom) in response to hypoglycemia (0.5 and 0 m m glucose). (**E**) Average secretion rates (in pC/min) during normoglycemia (5 m m glucose, basal) and in response to hypoglycemia (0.5 and 0 m m glucose) in WT mice and TH-Olfr78 KO mice. Data are represented as boxplots indicating median (middle line), 25th, 75th percentile (box), and largest and smallest values range (whiskers) with all individual data superimposed. *P*-values <0.05 or nearby values) calculated with Mann-Whitney test are indicated. Values in pC/min are basal (WT: 0.08 ± 0.04, *n* = 4 and KO, 0.3 ± 0.07, *n* = 5); 0.5 glucose (WT: 7.6 ± 1.4, *n* = 4 and KO, 3.2 ± 1.6, *n* = 3), 0 glucose (WT: 15.4 ± 5.7, *n* = 4 and KO, 10.5 ± 1.4, *n* = 5).

To further examine the neurosecretory responses of glomus cells in CB slices, we used amperometry to directly monitor the exocytotic release of dopamine (Figure 8A). This noninvasive assay allows to perform a quantitative evaluation of the intrinsic O_2_ sensitivity of individual glomus cells.^27,30^ Both WT and Olfr78-deficient cells responded with graded secretory responses to variable levels of hypoxia in the bath solution (hypoxia: ∼15 mmHg; 3% O_2_: ∼30 mmHg; 6% O_2_: ∼50 mmHg; control 21% O_2_: ∼150 mmHg) (Figure 8B and C). However, the secretory responses of Olfr78-deficient cells are smaller than those of WT cells across the range of O_2_ tensions tested; with differences statistically significant only for the lowest O_2_ tensions (Figure 8D-G). As the secretion rate depends not only on the exocytotic activity (frequency of events) but also on the charge carried by each secretory event, we quantified the quantal content of individual events in glomus cells that were exposed only to a low level of activation (O_2_ tension ∼50 mmHg) in order to prevent the fusion of vesicles in the cytosol before secretion (compound exocytosis) (Figure 8H). For context, we previously showed that glomus cells of HIF2α-deficient mice have a lower dopamine content per vesicle than WT cells.^14^ We found that Olfr78-deficient glomus cells also have a smaller mean charge per vesicle and a distribution of dopamine content in secretory granules displaced to smaller values compared to WT cells (Figure 8H and I). In addition to hypoxia, we tested the secretory response of these cells to challenges with high K^+^, hypercapnia and hypoglycemia, which are well-known glomus cell stimuli. Inappreciable differences between WT and Olfr78-deficient cells were seen for responses to high K^+^ and hypercapnia (20% CO_2_) (Figures 8B and 9A-C). However, there is a trend for Olfr78-deficient cells to show a lower response to hypoglycemia than WT cells (Figure 9D and E). Together, these data show that the absence of Olfr78 affects the neurosecretory function of CB glomus cells. Olfr78-deficient glomus cells have altered cytosolic Ca^2+^ dynamics, a reduction in dopamine content of secretory vesicles, and decreased secretory activity in response to hypoxia or hypoglycemia.

**Figure 10.**
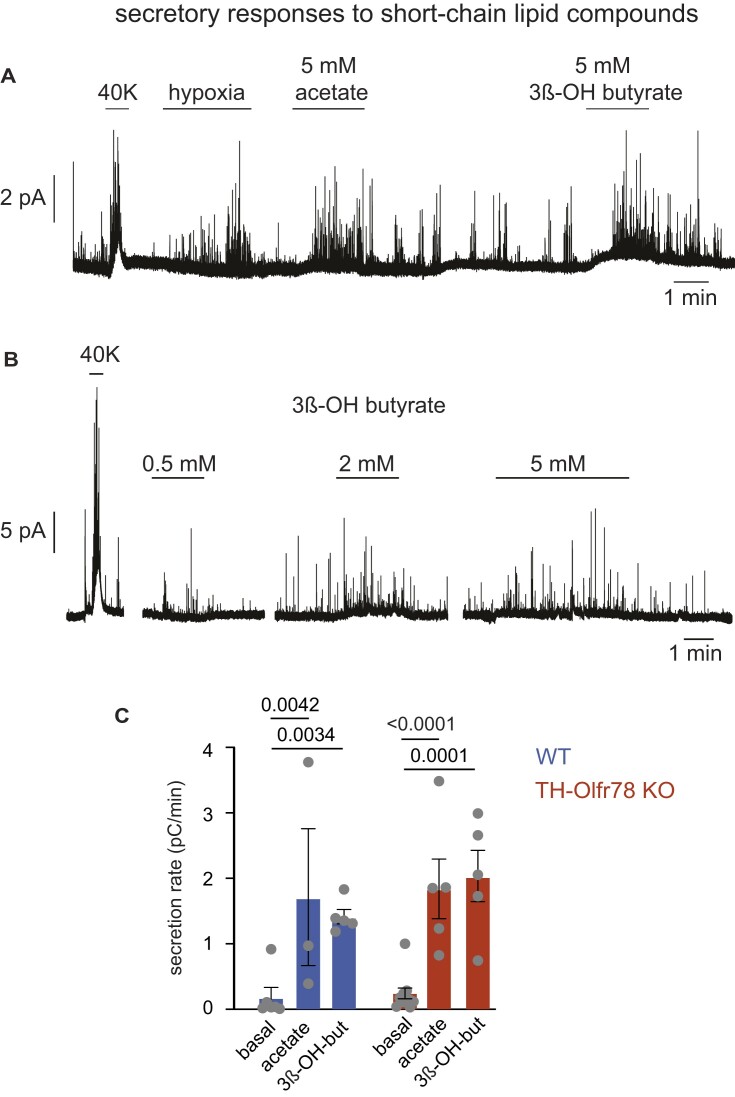
Secretory responses of glomus cells to short-chain lipid compounds. (**A**-**B**) Representative amperometric recordings illustrating glomus cell secretory responses to acetate and β-hydroxybutyrate in carotid body slices of TH-Olfr78 KO mice. (**C**) Quantification of the secretory responses of glomus cells to acetate (5 m m) and 3β-hydroxybutyrate (2-2.5 m m) in wild-type (WT) mice and TH-Olfr78 mice. Data are presented as bar diagrams indicating mean ± SEM. *P*-values (<0.05) calculated with parametric Dunnett’s multiple comparison test. Values in pC/min are WT (basal, 0.19 ± 0.15, *n* = 5; acetate, 1.7 ± 1, *n* = 3; 3β-hydroxybutyrate, 1.4 ± 0.1, *n* = 5); KO (basal, 0.24 ± 0.1, *n* = 5; acetate, 1.8 ± 0.4, *n* = 5; 3β-hydroxybutyrate, 2 ± 0.4, *n* = 5).

The *Olfr78* gene is ectopically expressed in several other tissues, such as the autonomic ganglia, kidney, and colon, where it has been suggested to confer responsiveness to short-chain fatty acids.^36–38^ Olfr78-deficient CB cells with robust responses to hypoxia and hypercapnia were also strongly activated by acetate or β-hydroxybutyrate, which are circulating metabolically relevant short-chain lipids (Figure 10A and B). Secretory activity induced by acetate or β-hydroxybutyrate was quantitatively similar in glomus cells of WT and TH-Olfr78 KO mice (Figure 10C).

## Discussion

The first conclusion of this study is that, as we showed previously,^7^ Olfr78 is not essential for the O_2_ regulation of breathing. Olfr78 is not a physiologically relevant lactate receptor^7,8^ and, despite its high level of expression in the CB, it is dispensable for both the HVR and CB growth during acclimatization to sustained hypoxia. Under our experimental conditions, we were not able to confirm the decrease in the ventilatory response to mild hypoxia (12% O_2_) reported in global *Olfr78* knockout mice.^8^ However, at the single glomus cell level, our results support previous observations indicating that CB sensory nerve firing is activated by short-chain lipid compounds, such as acetate or butyrate^8,39^ and that, as occurs with lactate,^7,12^ these responses are not affected in Olfr78 knockout mice.^8^

The second main conclusion of this study is that CB glomus cells of TH-Olfr78 KO mice exhibit in vitro complex phenotypic changes compared with WT cells. Although subtle in some respects, these are clearly identifiable. These changes included a trend for decreased *Th* expression, an increased expression of voltage-gated Na^+^ channels and altered neurosecretory responses consisting of a decrease in secretory vesicle dopamine content and of secretory responsiveness to hypoxia and hypoglycemia. Secretory responses to strong stimuli, such as high K^+^ or 20% CO_2_, are not significantly affected. We have also shown that, as in the case for other genes relevant to CB function,^14^  *Olfr78* mRNA expression is strongly downregulated in HIF2α-deficient CB cells.^15^ Therefore, although Olfr78 is not essential for acute O_2_ sensing, it appears to play a role in glomus cell homeostasis. Notably, the characteristics of Olfr78-deficient glomus cells resemble those of CB neuroblasts; these are immature glomus cell precursors that complete their maturation upon exposure to hypoxia.^17,40^ Carotid body neuroblasts are weakly TH-positive and have reduced dopamine quantal content and poor secretory activity and responsiveness to hypoxia but maintain responses to other stimuli. In addition, their capacitance (proportional to cell size) is smaller than that of glomus cells and more than 80% of these cells exhibit large Na^+^ currents, a sign of cellular immaturity.^17,41,42^ The similarities between Olfr78-deficient cells and CB neuroblasts suggest that Olfr78 exerts a “trophic” role that is required for the maturation of glomus cell phenotype; in this way, a deficiency of Olfr78 causes glomus cells to be in a more immature state. The Olfr78 deficiency may well be compensated for in vivo by other blood borne molecules acting on glomus cells in situ. However, CB glomus cells in in vitro preparations, subjected to enzymatic and/or mechanical stress and maintained for 24-48 h under culture conditions, may have a greater vulnerability to the lack of Olfr78-dependent trophic action. In this sense, it is also logical that the response to hypoxia—a complex process that depends on several steps, involving mitochondrial O_2_ sensing and signaling to membrane ion channels—in Olfr78-deficient cells is more affected than the secretion induced by direct depolarization with high extracellular K^+^. Moreover, the activation of glomus cells with strong stimuli (eg, 40 m m extracellular K^+^) favors compound exocytosis, thereby decreasing the number of small events (those more affected in Olfr78-deficient cells) and minimizing the effect of decreased quantal dopamine content on the total secretory activity.

The trophic action of Olfr8 may be related to the need of CB glomus cells to maintain high intracellular levels of cAMP. In addition to some well-known targets of cAMP-dependent transcriptional (CREB-mediated) and post-transcriptional (PKA-mediated) signaling pathways essential for glomus cell function, such as *Th* induction or Ca^2+^ channel phosphorylation, respectively,^43,44^ there may be many other targets that are yet to be elucidated. Indeed, genes encoding components of cAMP-dependent signaling pathways are among the most highly expressed in glomus cells.^2,3^ Furthermore, adenylate cyclase activation has a stimulatory action on glomus cells and potentiates the effect of hypoxia.^45^ Mice with glomus cells deficient in adenylate cyclase 3, a key component of the olfactory signal transduction pathway, exhibit impaired cellular and breathing responses to hypoxia.^46^ The physiological ligand(s) of Olfr78 is (are) unknown and the possibility that this G-protein-coupled receptor is constitutively activated^47^ cannot be discounted.

The contribution of Olfr78 to CB homeostasis suggests that its pharmacological modulation could be of therapeutic and translational interest. Exacerbation of the peripheral chemoreflex and sympathetic overactivation are involved in the pathogenesis of highly prevalent medical conditions, such as refractory hypertension, intermittent hypoxia during sleep apnea, and left cardiac failure.^30^ Remarkably, sympathetic activation and systemic hypertension induced by chronic intermittent hypoxia are reduced in constitutive Olfr78 knockout mice.^13^ As that chronic intermittent hypoxia activates the conversion of CB neuroblasts into mature glomus cells,^40^ an inhibition of this process could explain the decrease in sympathetic overactivation induced by intermittent hypoxia in constitutive (or global) Olfr78 knockout mice.^13^ In closing, we speculate that Olfr78 inhibitors, with a tissue or organ action that is more restricted to the CB than HIF2α inhibitors,^48,49^ may produce an attenuation of CB activity without entirely abolishing the life-saving responses to hypoxia and hypoglycemia.

## Data Availability

Data and materials may be requested by the corresponding authors.
